# Assessment of Drivers’ Perceptions of Connected Vehicle–Human Machine Interface for Driving Under Adverse Weather Conditions: Preliminary Findings From Wyoming

**DOI:** 10.3389/fpsyg.2020.01889

**Published:** 2020-08-18

**Authors:** Mohamed M. Ahmed, Guangchuan Yang, Sherif Gaweesh

**Affiliations:** Department of Civil and Architectural Engineering, University of Wyoming, Laramie, WY, United States

**Keywords:** Wyoming connected vehicle pilot, human–machine interface, driver behavior, human factors, driving simulator

## Abstract

Connected vehicle (CV) technology aims to improve drivers’ situational awareness through audible and visual warnings displayed on a human–machine interface (HMI), thus reducing crashes caused by human error. This paper developed a driving simulator test bed to assess the readability and usefulness of the Wyoming CV applications. A total number of 26 professional drivers were recruited to participate in a driving-simulator study. Prior to driving the simulator, the participants were trained on both the concept of CV technology and the developed CV applications as well as the operation of the driving simulator. Three driving simulation scenarios were designed. For each scenario, participants drove two times: one with the HMI turned on and another one with the HMI turned off. After driving the simulator, a comprehensive revealed-preference survey was employed to collect the participants’ perceptions of CV technology and Wyoming CV applications. Results show that the Wyoming CV applications were most favored under poor-visibility driving conditions. Among the Wyoming CV applications, forward collision warning and rerouting applications were experienced as the most useful. Approximately 89% of the participants stated that the Wyoming CV applications provided them with improved road condition information and increased their experienced safety while driving; 65% of the participants stated the CV applications and the HMI did not introduce distraction from the primary task of driving. Finally, this paper concludes that the design of CV HMI needs to balance a trade-off between the readability of the warnings and drivers’ capability to safely recognize and timely respond to the received warnings.

## Introduction

In the United States, Interstate 80 (I-80) is a major corridor for east–west freight movement and passenger travel in the country. The 402-mile I-80 freeway corridor in Wyoming is considered to be a unique freeway corridor because it is all located above 6,000 feet (1,829 m) in elevation and with very few alternate routes. As a mountainous rural freeway, the total traffic volume is not high; nevertheless, the commercial truck volume makes up 30–55% of the total traffic flow (WYDOT, 2017). As a consequence of Wyoming’s adverse winter weather conditions, such as snowstorms, strong crosswinds, icy road surface, and low visibility from blizzard and the presence of work zones, there have been remarkable traffic crash records along I-80 in Wyoming, which resulted in fatalities, road closures, and tremendous economic loss (WYDOT, 2017). In reality, it was found that more than 90% of motor vehicle crashes were attributed at least in part to human error (Nhtsa, 2015). With the booming of vehicle technology, connected vehicle (CV) technology has been widely introduced into the market at a fast pace. CV technology is designed to improve drivers’ awareness of hazards and situations they cannot even see through vehicle-to-vehicle (V2V), vehicle-to-infrastructure (V2I), and infrastructure-to-vehicle (I2V) dedicated short-range communication (DSRC) technologies so that proactive reactions could be made to avoid potential crashes (Shladover, 2018). A handful of studies have been conducted to assess the benefits of CV applications on reducing traffic collisions (Jeong et al., 2014; Dey et al., 2016; Olia et al., 2016; Zulkefli et al., 2017). In general, these studies demonstrate that CV technology has great potential in reducing the probability of traffic collisions on various transportation facilities and under different weather and traffic conditions.

With consideration of the challenging driving conditions on I-80 in Wyoming, the United States Department of Transportation (USDOT) selected Wyoming to develop, test, and deploy a suite of CV applications that utilize V2V, V2I, and I2V real-time communication technologies to provide warnings and advisories regarding various road conditions to heavy truck and light vehicle drivers (Gopalakrishna et al., 2016). The CV applications developed in the Wyoming CV pilot are expected to enable CV drivers to have awareness of upcoming hazardous traffic and roadway situations; therefore, drivers could make proactive reactions to avoid potential crashes. One of the key components of the Wyoming CV system is the on-board human–machine interface (HMI), which delivers received real-time geospecific basic safety messages (BSMs) and traveler information messages (TIMs) to drivers. Nevertheless, to date, there still lacks a clear understanding of how drivers recognize and response to the notifications displayed on the CV HMI. In fact, a well-designed HMI has the potential to provide CV drivers with proactive decision-making supports so that CV drivers could more timely respond to an imminent hazardous traffic condition and, thus, reduce the probability of involvement in traffic collisions. However, inappropriate integration of various CV warnings and advisories may mislead, distract, or even disturb drivers from their normal driving task (Li et al., 2017; Talamonti et al., 2017). These adverse effects are particularly significant during high-workload situations or driving under inclement weather and road surface conditions.

In this regard, this research aims to assess the effectiveness of the CV applications developed by the Wyoming CV Pilot Development Program. The assessment methodologies employed in this study have two steps. First, this research developed a CV driving-simulator test bed to simulate different traffic and weather conditions on I-80 in Wyoming. Then, professional snowplow truck and highway maintenance vehicle drivers from the Wyoming Department of Transportation (WYDOT) were invited to participate in the developed driving-simulator study. After experiencing the Wyoming CV applications in a simulated environment, each participant was requested to finalize a reveal-preference questionnaire survey, in which the participant provided perceptions of effectiveness of the CV applications as well as the visual distractions caused by the Wyoming CV HMI.

The remainder of this paper is organized as follows: Section “Literature Review” presents a review of the literature regarding HMI design and evaluation. Section “Description of Wyoming CV Applications and HMI” describes the functions of the Wyoming CV applications and HMI display layout. Section “Assessment of Wyoming CV HMI” documents the development of driving-simulator testing scenarios and participants’ evaluation of the CV applications after driving the developed simulation scenarios; finally, preliminary findings and discussion of the lessons learned from this pilot study are listed in Section “Concluding Remarks.”

## Literature Review

### HMI Display Design

In current practice, various modalities have been employed for the development of HMI display. In general, these modalities can be classified into four categories (Péter et al., 2017): mechanical, acoustic, visual, and haptic interfaces.

Mechanical interfaces require a mechanical interaction from the driver, which could be pressed by hand, finger, or foot; pulled, slid, or rotated by hand; or touched by hand or finger. The interfaces may include pedal, steering wheel, button, switch, stalk, slider, and controller knobs. Some advanced practices have been developed in these ordinary interfaces to enhance the driving performance on roads, such as electronic throttle control, electrical braking systems, electrical steering systems, etc. (Wang et al., 2016). Acoustic interfaces are common output interfaces because an acoustic (or auditory) interface does not require drivers to take off their eyes off the road; hence, it could be considered a safer modality than the visual one. These interfaces include beeps, voice feedback (i.e., spoken messages), and voice control. Beeps are suitable for drawing drivers’ attention. However, it provides unidentified information unless the driver recognizes the source of the beeper. Visual interfaces when used solely are usually used to communicate information in non-critical events. This is because visual messages could fail to deliver important information if the information displayed goes unnoticed by drivers. Over years, numerous visual interfaces were included in vehicles to suit different applications of autonomous and connected vehicles, including indicator lights, LCD displays, organic light emitting diode (OLED) displays, and head-up displays (HUD). However, the most detrimental effect of using visual interfaces is the possible increase in visual workload (Engström et al., 2015). The research also suggests that visual warnings could be used as supplemental information to an auditory or haptic warning. Haptic interfaces provide the driver with information through the driver’s tactile sense, such as a lane-keeping warning system that develops reaction torque when departing from the lane (Montiglio et al., 2006), and the haptic steering interface (Steele and Gillespie, 2001; Boyle, 2012), which can give navigation by developing sequenced pulses on the wheel clockwise or counterclockwise according to the required direction.

For the design format of messages that are displayed on the HMI, the Federal Highway Administration (FHWA) emphasizes that they should adhere to standard message formats. It is highly recommended to use familiar signs and messages that are similar to what is provided in the MUTCD (FHWA, 2015). This is because drivers may get confused with regard to the meaning of non-standard signs. In addition, spatial compatibility is required for the design of the message in the context of communicating information to drivers because the selection of a response is directly related to the position of the related stimulus. Information provided on HMIs should match what is provided on real-world traffic control devices (FHWA, 2015). Péter et al. (2017) point out that HMI devices are initially developed to provide services that enhance the efficiency of driving tasks. General aspects and standards for effective HMIs include the following requirements: readability, clarity, interpretability, accessibility, and ease of handling. Sentouh et al. (2014) indicate that the implementation of HMI should address a number of challenges, including what information is important for drivers, how information is displayed, when, under what circumstances, and in what order the information should be presented to drivers. Olaverri-Monreal and Jizba (2016) summarize the issues involved in the field of human–machine interaction; it was concluded that the in-vehicle HMI should provide an intuitively meaningful indication of the presence of a warning and its timely status. In addition, it is crucial to investigate driver distraction levels as well as the modality and dimension of the visual warnings and their suitable in-vehicle locations. Biondi et al. (2017) investigated the effectiveness of auditory, vibrotactile, and multimodal (i.e., combination of two or more modalities) HMI warnings; it was found that multimodal warnings appeared to be effective in low-workload conditions. However, the effect vanishes as the overall level of workload increases.

### Assessment of HMI

The most commonly used HMI design-assessment methodologies found in the literature are based on (1) stated-preference questionnaire surveys, (2) field-experiment testing using instrumented vehicles, and (3) driving-simulator testing.

For the questionnaire-survey method, Höltl and Trommer (2013) compared European drivers’ perceptions of advanced driver assistance systems (ADAS) through an online questionnaire survey, which aimed at collecting each driver’s rating of different ADAS applications in terms of perceived usefulness, ease of use, efficiency, and changed driving behavior. Bazilinskyy and de Winter (2015) conducted an international survey to gather drivers’ opinions and preferences on auditory interfaces. The results show that the auditory interfaces are preferred for the application of parking assistance and a forward collision warning (FCW) system. Another worldwide connected vehicle survey conducted by Accenture Consulting (2016) shows that traffic information, weather information, and a speed camera are the most popular HMI applications. For the field-experiment method, Fitch et al. (2014) investigated whether collision avoidance systems should present individual crash alerts in a multiple-conflict scenario or present only one alert in response to the first conflict. This was because, in reality, secondary alerts may startle, confuse, or interfere with drivers’ execution of an emergency maneuver. Testing results show that drivers who received both the FCW and lane-change merge alerts were significantly faster at steering away from the lateral crash threat than the drivers who received only the FCW alert. Song et al. (2016) evaluated drivers’ response to HMI under two different types of warning systems, emergency warning and general warning, by combining various modalities. Study results show that, for emergency alerts, the most effective warning information was transmitted by integrating “voice, graphic, and text” or “repeated computer tone and text.” In the case of a general warning alert, the “repeated computer tone, voice, graphic, and text” combination was indicated to be the most effective.

Bao et al. (2012) evaluated truck drivers’ following behavior to an in-vehicle crash warning system in a naturalistic driving environment. Results indicate that the presence of warnings increased mean time-headway by 0.28 s, and drivers’ response time to the forward collisions was 15% faster than the baseline condition (i.e., no in-vehicle crash warning system). Biondi et al. (2018) developed a rating tool for assessing HMIs of various ADASs. Based on a field-experiment testing, the authors point out issues that are related to visual, auditory, and haptic warnings; for example, auditory warnings used by the rear parking sensor were not indicative of the distance of the vehicle to obstacles, visual warnings adopted by a blindspot monitor were located in unconventional locations, and accelerations operated by the lane keep assist system were in some cases uncomfortable and jolty.

In comparison with the questionnaire survey and field-experiment methods, a driving simulator has the advantages of testing different HMI design alternatives in a safe environment, and environmental variables can be better controlled. Cummings et al. (2007) investigated the impacts of single versus multiple warnings on driver performance. It was found that participants’ reaction times and accuracy rates were significantly affected by the type of collision event and alarm reliability. Moreover, the use of individual warnings did not significantly affect driving performance in terms of reaction time or response accuracy. Osman et al. (2015) tested the location of the visual HMI display in a connected-vehicle simulator experiment. Results reveal that the majority of respondents preferred the visual display to be provided as a HUD in the midsection of the windshield. Jakus et al. (2015) investigated the effectiveness of integrating multimodal interfaces and using single-modal interfaces. Three different interfaces were defined: (1) visual, (2) auditory, and (3) a multimodal auditory and visual interface. Results show that the interaction with visual and audio head-up displays was significantly faster and safer. In term of efficiency, no significant difference was found between the visual only and audiovisual modalities. However, the majority of the users preferred to use multimodal interfaces. Zhao et al. (2016) developed an integrated driving simulator and microsimulation modeling framework to evaluate the environmental benefits of CV applications. The authors point out that driving simulator–based experiments have the advantage of accounting for the response of human drivers to the recommended speed profiles, thus safely and more accurately evaluating the benefits of CV applications. Ma et al. (2016) employed a driving simulator to compare the effectiveness of physical roadside dynamic message signs (DMS) and virtual DMS (VDMS) generated by CV technology. Effectiveness was measured in terms of message comprehension, distraction, and overall difficulty level in receiving messages. It was concluded that, in general, VDMS performed better than DMS, particularly with the increase of the message length and under higher driving workload conditions. Schwarz and Fastenmeier (2017) investigated the effects of modality (e.g., auditory vs. visual) and specificity (e.g., low vs. high volumes) on warning effectiveness. Results show that the effects of specificity is dependent on the modality of the warning. Francois et al. (2017) compared three speedometer display patterns in a simulated truck-driving setting: digital, analog, and redundant speedometers. It was found that the digital speedometer is more efficient and less visually distracting for absolute and relative reading tasks, whereas the analog speedometer is more effective for detecting a dynamic speed change. The redundant speedometer has the best performance when compared to the two single types for each of the three reading tasks. Naujoks et al. (2017) explored the potential of using visual-auditory HMI to inform drivers in a non-distracting way. Based on the driving-simulator testing, it was found that participants clearly favored the HMI with additional speech-based output, which demonstrates the potential of speech to enhance the usefulness and acceptance of HMI. Houtenbos et al. (2017) examined the effects of audiovisual warning of the speed and direction of intersecting vehicles at intersections using a driving simulator. Based on a postexperiment questionnaire survey, the authors conclude that the beeps (audio modality) were regarded as more useful than the lights (visual modality). Vaezipour et al. (2018) designed a driving simulator experiment to investigate drivers’ acceptance of various types of in-vehicle HMIs (i.e., visual advice only, visual feedback only, and visual advice plus feedback) and the impact of in-vehicle HMI on driving behavior. Results show that visual advice only HMI was most accepted by participants, and both advice and feedback HMIs were found to benefit eco-safe driving behavior.

With consideration of the costs of each assessment methodology and the availability of facilities at the time the research was conducted, this research employed integrated driving-simulator testing and revealed-preference questionnaire survey methods to identify drivers’ perceptions of Wyoming CV HMI.

## Description of Wyoming CV Applications and HMI

### CV Applications

The Wyoming CV applications were classified into five categories based on their function and communication technologies (Gopalakrishna et al., 2016): category 1: forward collision warning (FCW), category 2: distress notification (DN), category 3: situational awareness (SA), category 4: work zone warnings (WZW), and category 5: spot weather impact warning (SWIW). A detailed illustration of the existing communication and traffic control devices along the Wyoming I-80 corridor and the DSRC locations that are deployed on the corridor can be found in Ahmed et al. (2019a).

#### Forward Collision Warning (FCW)

Forward collision warning is a V2V communication-based safety application that issues a warning to the CV driver in case of an impending front-to-rear collision with another CV ahead in traffic in the same lane and direction of travel. FCW aims to help CV drivers avoid or mitigate front-to-rear vehicle collisions in the forward path of travel. This CV application is critically important for safety along I-80 in conditions when snowplows are moving slower than following traffic and/or low visibility conditions caused by adverse weather. The developed FCW has two warning levels: the cautionary level and the alert level. The HMI displays a yellow cautionary warning icon along with a loud beep sound when the time-to-collision is greater than 5 s but less than 9 s; drivers need to be prepared to brake when receiving the cautionary FCW. When the collision time is less than 5 s, the alert FCW is triggered; the HMI displays a red warning icon along with continuous loud beeps. Drivers need to immediately begin braking to avoid rear-ending the leading vehicle.

#### Distress Notification (DN)

Distress notification is a V2I communication-based safety application that enables CVs to communicate a distress status back to the Wyoming CV system when the vehicle’s sensors detect an event that might require assistance from others (e.g., air bag deployed and vehicle disabled) or the CV driver manually initiates a distress notification. The DN, which includes the vehicle category, location, content, and time of the message, is sent to the nearest roadside unit (RSU). The RSU forward it to the Wyoming CV system for notifying system operators. If an RSU is out of the range of DSRC, the DN is expected to be received by nearby CVs that are traveling in the same and/or opposite direction via V2V communication. These CVs will forward the DN to an RSU that is connected to the Wyoming CV system.

#### Situational Awareness (SA)

The SA application adopts I2V and V2I communication technologies to assemble important travel information from Wyoming CV system operators and communicate them directly to CV drivers through both DSRC and satellite communications. SA enables delivery of up-to-date downstream traffic and road conditions that may affect driving safety to CV drivers. The SA application includes weather alerts, speed limitations, vehicle restrictions, road surface conditions, incidents ahead advisories, truck parking availability, and road closures, etc.

#### Work Zone Warnings (WZW)

The WZW application employs I2V communication technology to provide CV drivers information about the unsafe conditions that exist in an active work zone, such as obstructions in the vehicle’s travel lane, lane shifts and closures, speed reductions, and construction vehicles/workers entering or exiting the work zone.

#### Spot Weather Impact Warning

SWIW is a special case of SA that warns CV drivers of local hazardous weather conditions, such as rain, snow, fog, or strong winds. The primary difference between SWIW and other SA applications is that it provides more localized information.

The majority of the visual warnings were developed following Manual on Uniform Traffic Control Devices (MUTCD) guidance (FHWA, 2009); detailed descriptions of each CV application, including its communication technology or technologies, visuals that are displayed on the CV HMI, and messages delivered by this CV application, are summarized in Table 1.

**TABLE 1:** Summary of the Wyoming CV applications.

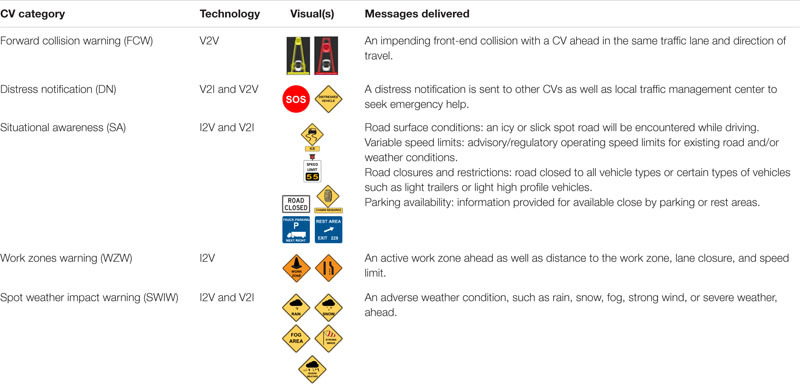

### Layout of Wyoming CV HMI

**FIGURE 1:**
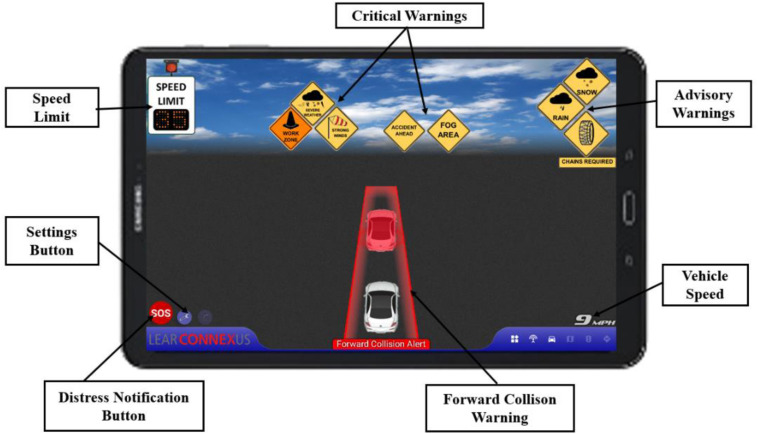
Layout of the Wyoming CV HMI (Ahmed et al., 2019b).

Figure 1 illustrates how the Wyoming CV warnings are displayed on the HMI screen. In general, these CV warnings are categorized into four priority levels based on the urgency of the imminent situation: Level 1–FCW, Level 2–variable and regular speed limit, Level 3–critical warnings, and Level 4–advisory warnings, respectively. In this pilot study, critical warnings were determined to be situations that would significantly affect driver’s operation of vehicle (e.g., icy road surface, work zone, severe weather, etc.) or appear beyond expectation (e.g., road closure, accident or distressed vehicle ahead, fog or strong wind ahead, etc.). Advisory warnings aimed to provide advisory information to draw drivers’ awareness while driving, mainly including adverse weather conditions that may affect driving, such as rain and snow, location of rest area or parking area, etc. Within the critical and advisory warnings, in case there are multiple warnings appearing simultaneously on the HMI, warnings that are more urgent are displayed closer to the driver, i.e., on the left side of the HMI.

## Assessment of Wyoming CV HMI

### Apparatus

**FIGURE 2:**
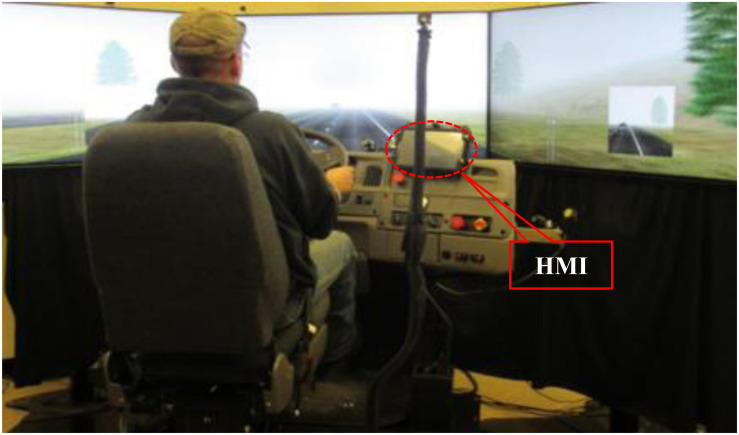
Truck simulator with CV HMI.

The CV driving simulator study was conducted at the University of Wyoming Driving Simulator Lab (WyoSafeSim). The motion-based, high-fidelity driving simulator can switch between a passenger car (2004 Ford Fusion) cockpit cab and a freight truck (2000 Sterling AT9500 18-wheeler semi-trailer) cockpit cab. It is mounted on a three-degrees-of-freedom D-Box motion platform, which comprises four electro-mechanical linear actuators to provide two rotational and one translational degrees of freedom (roll, pitch, and heave). The simulator provides motion cues to immerse the driver into a real driving experience with changes in kinematics, such as velocity, acceleration, and deceleration. In addition, a low-frequency vibration transducer is mounted on the vehicle floor to simulate vibrations generated by engine and road. The simulator has open architecture software with complete source code of simulation creator tool, which offers flexibility of building roadways and developing driving scenarios that could replicate the actual driving environments. The CV HMI was mounted on the dashboard of the simulator to provide participants with the various CV warnings as illustrated in Figure 2.

### Participants

This research recruited a total of 26 professional drivers to participate in the CV driving-simulator study to assess the effectiveness of the Wyoming CV HMI. The participants are professional snowplow truck drivers and employees from WYDOT; they are expected driving connected trucks and connected maintenance vehicles after the full deployment of the CV system in Wyoming. The selection of participants considered a wide range of factors that might affect the acceptance or perception of CV technology, such as age, education level, driving experience, etc. Because potential Wyoming CV users are commercial truck drivers, WYDOT snowplow truck and freeway maintenance vehicle drivers, and Highway Patrol vehicle drivers, at this stage, all the participants were male. Based on a predrive survey questionnaire, it was summarized that the participants’ ages ranged from 21 to 61 years (Mean = 42; *S.D*. = 10.3). Among the 26 participants, 15 graduated from high school, nine have a college degree, and two have a postgraduate degree. All participants had a valid commercial or class C driver’s license with an average driving experience of 14.5 years (range: 0.5–36 years, *S.D*. = 11). Twenty-five of the participants reported they never had any ophthalmic surgery (one participant had laser vision correction in 2006). During the driving simulator study, all the participants were in good health condition without vision, audition, and emotional issues that might affect their normal driving (e.g., angry, depressed, dizzy, etc.). All the participants reported that they have encountered reduction in visibility due to snow, blizzards, fog, smoke, or heavy rain while driving on I-80 in Wyoming.

### Driving-Simulator Study Scenarios

**FIGURE 3:**
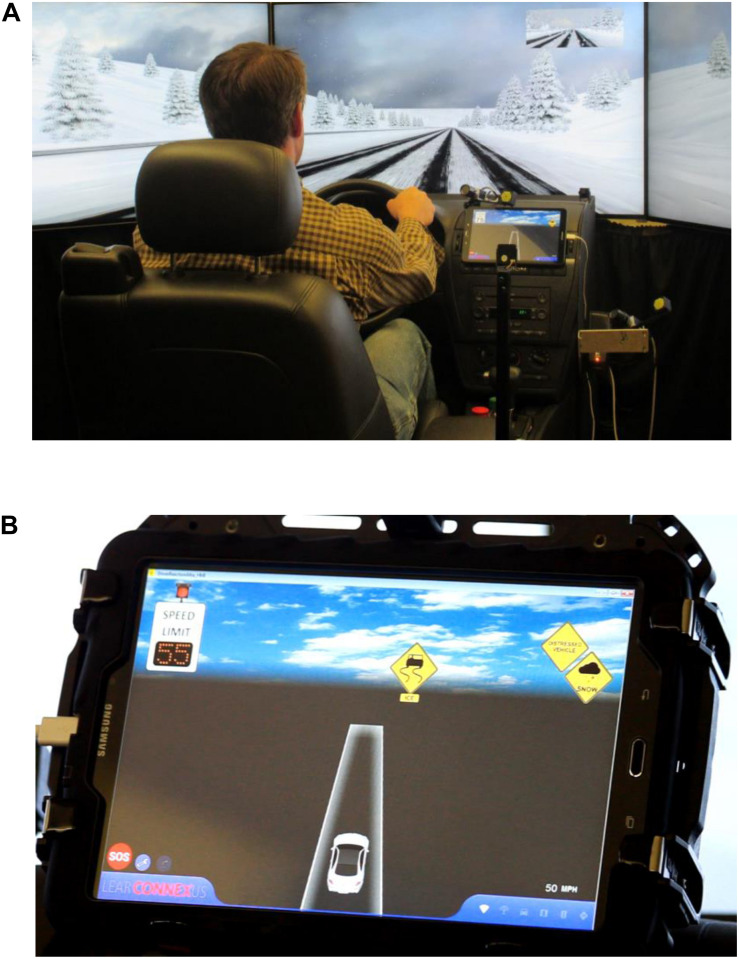
Illustration of driving simulator study at WyoSafeSim lab: **(A)** A simulation scenario; **(B)** CV HMI.

Three comprehensive simulation scenarios were developed to simulate different real-world traffic and weather conditions on I-80-like freeways: work zone with FCW in fog, slippery road surface due to snowy weather, and road closure due to accident in severe weather, respectively. After a warm-up session, each participant drove each simulation scenario two times; one with the CV HMI turned on and the other one with the CV HMI turned off. To eliminate the potential impact of any learning effect on the simulation result, this research randomly assigned the sequence of these six simulation scenarios to each participant. Prior to the driving simulator study, participants were provided with training on both the basic concept of the Wyoming CV system and hands-on operation of the driving simulator under the CV environment. Figure 3 illustrates the driving simulator study at the WyoSafeSim lab.

The driving simulator test bed was designed as a two-way, four-lane freeway segment with a 75-mph (120 km/h) speed limit to represent the basic operational conditions of I-80 in Wyoming. To control for the potential impact of the ambient traffic on participants’ driving behavior, the average and standard deviation of speed of the ambient traffic was coded to match speed distributions similar to the Wyoming I-80 in alike adverse weather conditions.

The work zone simulation scenario aimed to test the Wyoming CV system’s WZW and FCW applications. These CV applications are expected to help in avoiding potential collisions at a freeway work zone due to reduced visibility caused by fog. The general simulation procedure is detailed as follows:

Participants first accelerated to the normal freeway driving speed (i.e., 75 mph). A fog area was design ahead of a work zone; a “fog area” CV warning with an advisory speed limit of 65 mph (105 km/h) were displayed on the CV HMI before participants entered the fog area. In the work zone, the right lane of the freeway was closed following typical construction zone layouts in Wyoming; a series of WZWs along with an advisory speed limit of 45 mph (75 km/h) were shown on the CV HMI to alert participants to change lanes and reduce speed before entering the work zone. To simulate an FCW, a slow-moving truck was designed to appear in the work zone; a worker suddenly crossed the lane in front of the slow-moving truck, and thus, the truck made an emergency brake to yield to the worker. A proximity sensor was employed to trigger the truck, indicating that the truck could make the braking action at the designated distance in front of the simulator vehicle. Then, with V2V communication technology, an advisory and an alert FCW were displayed successively on the CV HMI to notify participants of the potential forward collision. It is worth mentioning that the foggy condition was created to allow a safe stopping sight distance for 45 mph for the simulator vehicle type, i.e., heavy truck.

The slippery road surface simulation scenario was designed to test the Wyoming CV system’s SWIW and DN applications. Functions of these CV applications were to warn the participants to reduce speed before entering an icy road segment, thus avoiding skidding off the travel lane or being involved in a secondary crash.

This simulation scenario started with a snowy weather condition; a “snow” CV warning with an advisory speed limit of 65 mph appeared on the CV HMI. Later on, a “severe weather” CV warning with an advisory speed of 45 mph were displayed on the CV HMI. Before entering the icy road segment, an “icy surface” CV warning with a 35 mph (55 km/h) advisory speed limit were displayed on the CV HMI to warn participants to reduce speed when driving on the icy road. Prior to entering the icy curve, a “distressed vehicle” warning was received to alert participants there was a skidding-off accident ahead, indicating that participants should drive with extreme caution. For participants who lost control of the vehicle due to speeding, they were asked to use the DN application to generate and send a distress message to the TMC and other CVs on the road (after sending the DN, the simulation scenario was automatically terminated).

The road closure simulation scenario intended to test the Wyoming CV system’s SWIW and SA applications. These CV applications provided participants with real-time road closure notification due to an incident as well as information about the nearest rest area to help participants avoid being jammed on the closed freeway or involve in a secondary crash. A “snow” CV warning with a speed limit of 65 mph was displayed on the CV HMI. Later on, a “severe weather” CV warning with an advisory speed of 45 mph were displayed. A pile-up crash was designed on the freeway mainline to simulate a road closure condition; the crash was located downstream of a rest area. “Accident ahead” and “road closed” warnings were displayed on the CV HMI; then, a “rest area” notification appeared on the CV HMI to inform participants about the nearest rest area. If a participant exited the freeway to the rest area, a voice message was played to inform the participant to park the vehicle and stop this driving simulator scenario. Otherwise, the participant was queued in front of the crash location on the freeway.

**FIGURE 4:**
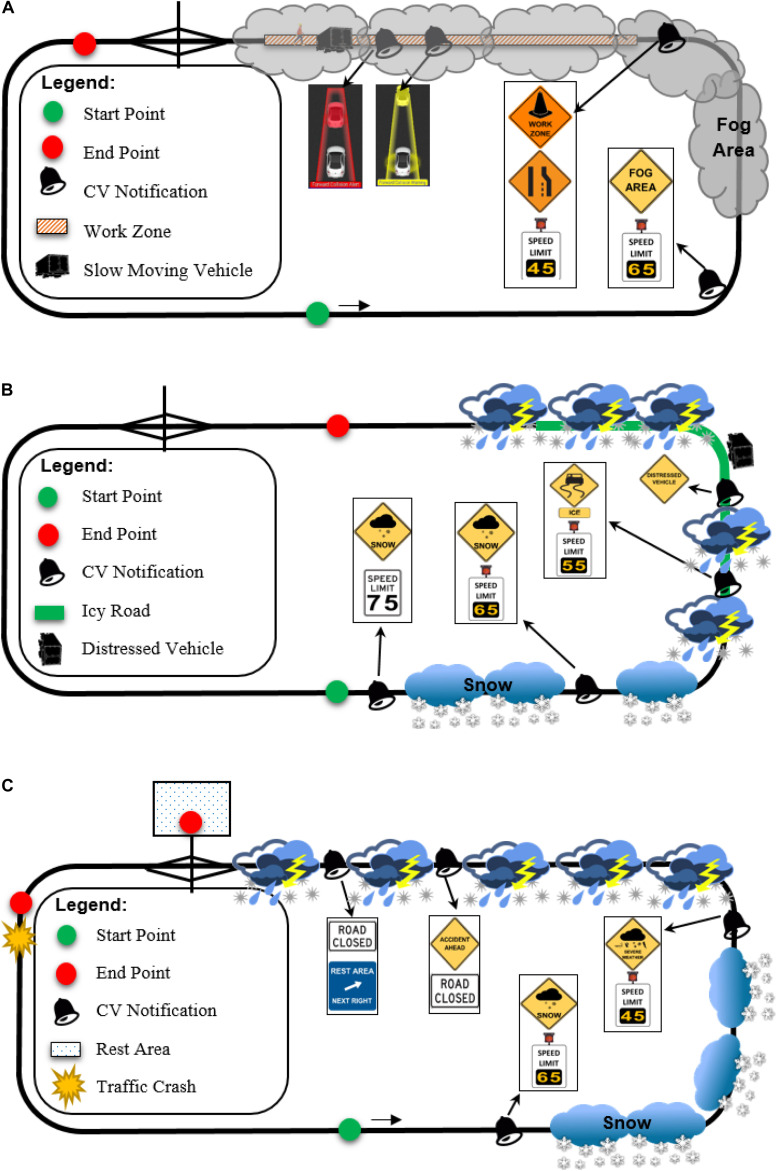
Description of driving simulator study scenarios: **(A)** work zone with FCW in fog; **(B)** slippery road surface due to snowy weather; **(C)** road closure due to accident in severe weather.

Sequence of the CV warnings and general layout of each driving-simulator study scenario are illustrated in Figure 4.

### Questionnaire Survey

After experiencing the Wyoming CV application in the driving-simulator study, a comprehensive postdrive questionnaire survey was employed to collect participants’ qualitative opinions regarding their preferences on different CV warning modalities and the effectiveness of CV technology under various real-world driving conditions. The questionnaire survey was initially designed by the University of Wyoming research team and then reviewed, revised, and approved by the USDOT Volpe National Transportation Systems Center.

**FIGURE 5:**
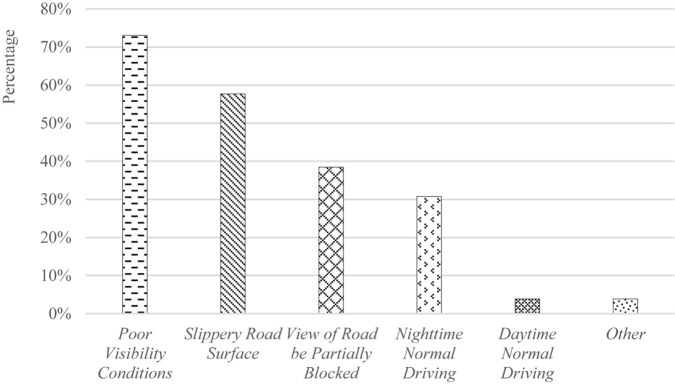
Rank of the effectiveness of CV technology under various driving conditions. Poor visibility conditions = rain, fog, snow, sun glare weather conditions; view of road be partially blocked = view of the road ahead is partially blocked by other vehicles or the curves and other terrains; other = road work.

Results show that the majority of participants (96.2%) preferred to have the CV warnings displayed at the combination of visual and auditory modalities. For the auditory-warning modality, it was found that using a simple “beep” sound for advisory warnings and a series of louder “beep” sounds for critical warnings would best draw a driver’s attention while a repeated voice message tended to disturb normal driving. For the visual-warning modality, results show that, by grouping CV warnings to different priority levels and presenting warnings that have a higher priority closer to the driver (i.e., left side on the CV HMI), drivers tended to more easily perceive the imminent safety hazard when multiple warnings were displayed on the CV HMI. Overall, participants indicated that CV technology was most useful under poor-visibility driving conditions, such as rainy, foggy, blowing snow, and sun glare weather conditions, as illustrated in Figure 5. It was found that, under normal daytime driving conditions, participants felt that CV technology did not introduce significant benefits in comparison with when driving under adverse weather conditions that resulted in a slippery road surface, when the view of the road ahead was partially blocked by other vehicles on the curvy terrain, or driving at night.

In addition to qualitative descriptions, the questionnaire survey also collected participants’ assessment of the readability and usefulness of the Wyoming CV applications. Readability refers to how easily participants felt they recognized a CV warning or a bundle of CV warnings; usefulness means whether a CV warning helped drivers to recognize an imminent safety hazard or assisted them in better planning their trip. The assessment contains two components: assessment of CV technology and the specific CV applications, respectively. Responses were measured on a 7-point Likert scale (example: strongly disagree to strongly agree). Accordingly, the evaluation results were converted to a 1–7 score, in which score 1 corresponds to a very negative assessment result and score 7 to a very positive assessment result. The numerical values were used for quantification of assessments and comparisons across different CV applications.

Table 2 presents 26 participants’ assessment results of the Wyoming CV system. In addition to the scores generated from the Likert scale questionnaire survey, this paper categorizes participants’ perceptions of CV technology into three categories: positive (scores 5–7), neutral (score 4), and negative (scores 1–3). In general, the majority of participants provided positive feedback regarding the Wyoming CV applications and indicated that CV technology provided improved road condition information and would help to increase traffic safety.

**TABLE 2:** Participants’ assessment results of CV technology.

**Scale items**	**Mean**	***SE***	**Positive**	**Neutral**	**Negative**
**(a) Readability of CV warnings**					
A1: After experiencing the CV applications, how easy was it to understand the CV technology and warnings?	6.1	0.80	96.2%	3.8%	0%
A2: Do you think that warnings among the different CV applications are confusing?	5.5	0.95	80.8%**	19.2%	0%
A3: Do you think that the CV warnings and the display unit are introducing any distraction from the main driving task?	5.2	1.37	73.1%***	15.4%	11.5%
A4: Were the visual warnings clear, obvious, and convey the required message?	5.7	0.93	84.6%	15.4%	0%
**(b) Usefulness of CV Technology**					
B1: Do you think the CV system provided you with improved road condition information?	5.8*	1.14	85.7%	9.5%	4.8%
B2: Do you think that having the CV applications would help to increase traffic safety and reduce crashes?	5.9	1.14	88.5%	7.7%	3.8%
B3: How likely will you be dependent on the CV applications to warn you for upcoming hazardous conditions, when fully implemented on I-80?	4.2	1.61	42.3%	30.8%	26.9%
B4: Would you like to have the CV applications in your vehicle?	4.8	1.67	65.4%	23.1%	11.5%

*Based on 21 available samples; **positive feedback means CV applications were NOT EXPERIENCED AS confusing; ***positive feedback means CV applications and the display units were not experienced as introducing any distraction.

Table 3 presents participants’ assessment results of the readability and usefulness of the specific CV applications. Overall, the readability and usefulness of the Wyoming CV applications have been well accepted by the participants; specifically, FCW and rerouting notifications were found to be most useful.

**TABLE 3:** Participants’ assessment results of the specific CV applications.

**CV applications**	**Readability**					**Usefulness**				
	**Mean**	***SE***	**Positive**	**Neutral**	**Negative**	**Mean**	***SE***	**Positive**	**Neutral**	**Negative**
FCW	5.9	0.99	84.6%	15.4%	0%	6.1	1.03	88.5%	11.5%	0%
DN	6.0	1.11	88.5%	7.7%	3.8%	5.7	1.12	84.6%	11.5%	3.8%
SA (road surface)	6.1	0.80	96.2%	3.8%	0%	5.7	1.08	84.6%	11.5%	3.8%
SA (rerouting)	6.1	1.13	92.3%	3.8%	3.8%	6.0	1.10	84.6%	15.4%	0%
WZW	6.2	0.97	88.5%	11.5%	0%	5.8	1.24	80.8%	15.4%	3.8%
SWIW	5.9	0.91	92.3%	7.7%	0%	5.5	1.27	73.1%	19.2%	7.7%

## Concluding Remarks

This study assessed the subjective experiences related to the readability and usefulness of the Wyoming CV application in a simulated environment. It was found that the majority of the participants preferred to have the CV warnings provided in a combination of visual and auditory modalities. For visual warnings, this study grouped the CV warnings into four priority levels and presented warnings that have a higher priority closer to the driver. This was considered by the participants to be an effective way for them to perceive the imminent safety hazard when multiple warnings were displayed on the HMI simultaneously. For auditory warnings, the participants reported that a simple “beep” sound for advisory warnings and a series of louder “beep” sounds for critical warnings would best draw their attention while a repeated voice message tended to disturb normal driving. The participants indicated that CV technology was most useful under poor-visibility driving conditions; FCW and rerouting were the most useful CV applications. It is worth pointing out that FCW and rerouting CV applications have the most significant potential to realize the WYDOT CV pilot’s strategic goals to improve safety and mobility. Generally speaking, FCW and rerouting applications are tactical-level CV applications, which can directly help drivers to avoid a crash or being congested on the freeway. In comparison, DN, SWIW, WZW, and other SA applications are strategic-level CV applications, which aim to assist drivers more easily to recognize safety hazards or unexpected events, particularly when drivers’ recognition ability is limited by visibility.

Nevertheless, assessment results reveal that there are still a couple of issues that need to be considered to further improve the design of the Wyoming CV HMI. The primary issue is the potential distraction of CV HMI. As presented in Table 2, approximately 27% of participants indicated that distraction could be introduced by the Wyoming CV HMI (i.e., 12% found the CV warning distracting, and 15% found them neutral). Another issue is that the usefulness of CV technology tends to be less significant during normal daytime driving conditions or when drivers can recognize hazardous conditions without receiving CV warnings. From Table 2, it was found that only 42% of participants stated that they are going to depend on the CV applications to identify upcoming hazards, and less than two thirds of the participants showed desirability of having CV technology in their vehicles. These findings are consistent with a previous study that found drivers may not exactly trust in advanced driver assistance systems (Kidd et al., 2017) and also further proved previous research findings that truck drivers would like to receive acceptable feedback that is designed and implemented properly (Roetting et al., 2003) and displeasure with the continuous auditory warnings (Bazilinskyy et al., 2019). Therefore, these findings indicate that, under normal daytime driving conditions, the repeated auditory or visual CV warnings might distract drivers from their driving task. With this consideration, this study suggests that the design of CV HMI needs to add a user customization capability to suit the needs of individual users, such as a CV system that can be automatically or manually deactivated under normal daytime driving conditions. Nevertheless, it is necessary to clarify that this study is highly practice-focused, which aimed at supporting the WYDOT CV pilot. At this stage, this study only recruited 18 male drivers from WYDOT and the trucking industry; findings of this pilot study presented some preliminary insights into the optimal design of CV HMI display in a way that drivers can perceive CV warnings promptly without being distracted. Considering the increasingly popularity of CV technology, future studies need to recruit a larger number of participants that cover a wider range of demographic features to further investigate general drivers’ perceptions of the Wyoming CV HMI through statistical analysis and modeling, which will further benefit the design of CV HMI for general purposes (Engström et al., 2015; Biondi et al., 2017; Vaezipour et al., 2018, 2019).

In fact, safe driving is the principle task for human drivers. As specified by the National Highway Traffic Safety Administration (Nhtsa, 2009), the primary requirement of the in-vehicle HMI is to deliver desired warnings or notifications to a driver while minimizing driver distraction. Therefore, the optimal design of CV HMI needs to balance a trade-off between the readability of the messages (e.g., maximum number of messages displayed on the CV HMI, length of each message and the modality of the message, etc.) and drivers’ capability to safely recognize and timely respond to the received message(s). This is particularly critical during high-workload situations or under adverse weather conditions when drivers need more response and reaction time to an unexpected event because overloaded CV HMI information may distract the driver and lead to safety issues. However, there is still a lack of a comprehensive assessment of the effectiveness of different CV HMI display designs and development of CV HMI design guidelines considering human factors. Specifically, the following aspects need to be further investigated: (1) Which kind of HMI display modality (i.e., visual, auditory, voice message, or a combination of visual and auditory) best delivers the meaning of a warning? (2) What is the maximum number of warnings that can be displayed on the HMI without confusing drivers? (3) When should an early warning be displayed and how long should the warning remain on the HMI? (4) How to prioritize different warnings when they are displayed simultaneously on the HMI. In summary, incorporating human factors into the design and development of CV HMI has become increasingly critical, which aims to minimize the potential distractions introduced by these in-vehicle technologies.

## Data Availability Statement

The data that support the findings will be available upon reasonable request from the corresponding author, MA.

## Ethics Statement

The studies involving human participants were reviewed and approved by University of Wyoming IRB. The patients/participants provided their written informed consent to participate in this study.

## Author Contributions

MA, GY, and SG: study conception and design. SG and GY: experiments and data collection. GY: analysis and interpretation of results. GY and MA: drafted manuscript preparation. All authors reviewed the results and approved the final version of the manuscript.

## Conflict of Interest

The authors declare that the research was conducted in the absence of any commercial or financial relationships that could be construed as a potential conflict of interest.
